# The multifaceted role of lactate in cardiovascular health: from metabolism to signaling and epigenetics

**DOI:** 10.1080/15592294.2025.2576069

**Published:** 2025-10-29

**Authors:** J. Konieczny, N. Boardman, E. Aasum, A. Hafstad, L. Hortells, S. Geiseler

**Affiliations:** Cardiovascular Research Group, Department of Medical Biology, Faculty of Health Science, Uit - The Arctic University of Norway, Tromsø, Norway

**Keywords:** Lactate, lactylation, HCAR1, cardiovascular

## Abstract

Lactate, long regarded as a metabolic byproduct of anaerobic glycolysis, has emerged as a key regulator of cardiovascular health. Its roles extend beyond energy metabolism and include cell signaling through hydroxycarboxylic acid receptor 1 (HCAR1/GPR81) and epigenetic regulation via lactylation. These interconnected mechanisms influence diverse processes in the cardiovascular system such as energy production, angiogenesis, inflammation, and fibrosis. In this review we provide a chronological exploration of lactate’s functions, focusing on its role in cardiovascular physiology and with a particular emphasis on its role in epigenetics, highlighting the connecting points among these mechanisms and proposing areas for future research.

## GRAPHICAL ABSTRACT

**Figure:**
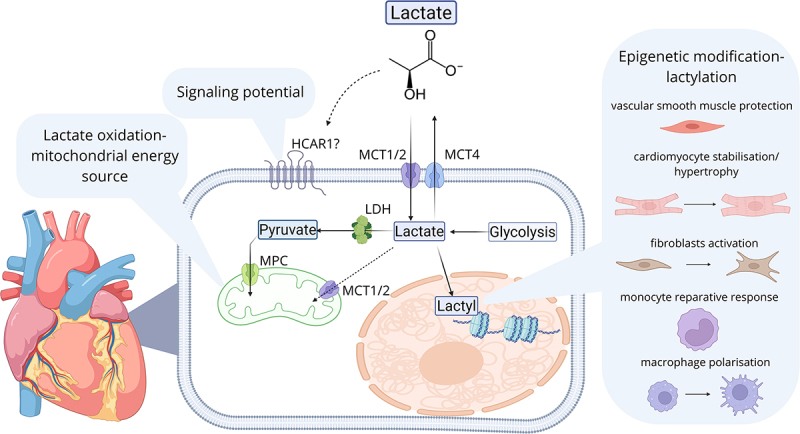
**Graphical abstract:** The multifaceted role of lactate in cardiovascular health - schematic representation of the mechanisms of lactate’s extra- and intra-cellular transport, followed by oxidation and usage as energy source, as well as lactyl group creation resulting in epigenetic marking. Exogenous lactate can enter the cell through monocarboxylate transporter 1 and 2 (MCT1/2) and be transported out by monocarboxylate transporter 4 (MCT4). Signaling through hydroxycarboxylic acid receptor 1 (HCAR1), has been shown in other organs including skeletal muscle, but remains to be elucidated in the cardiovascular system. Once in cytosol, or produced in glycolysis, lactate can undergo oxidation by lactate dehydrogenase (LDH) and act as a robust energy source, when reduced to pyruvate, as it enters mitochondria via mitochondrial pyruvate carrier (MPC). Findings in the skeletal muscle indicate a potential alternative path of transport via mitochondrial membrane MCT’s and oxidation of lactate within mitochondria by mitochondrial LDH, but this has not been validated in the heart yet. Cytosolic lactate can also act as an epigenetic marker, both in histones and non-histone proteins. Lactylation has been reported to exert an effect on a variety of cells in cardiovascular system. Image created with BioRender.com

## Introduction

Most people know lactate from the burning sensation that accompanies strenuous exercise, where it is often framed as a ‘waste product’ of anaerobic glycolysis. In reality, lactate is a dynamic metabolite that circulates continuously at rest and during activity, linking tissues and organ systems and is starting to be recognized as a versatile regulator in physiology [1]. In the heart, it serves as a major oxidative fuel during exercise or stress and is distributed by monocarboxylate transporters (MCT) that shape intracellular availability [1,2]. As a signal, lactate activates the lactate G-protein coupled receptor HCAR1, promoting adaptive processes such as angiogenesis and tissue protection [3]. Recent advances in the field of epigenetics have led to the discovery of lactate acting as an epigenetic modifier by process of lysine lactylation on histone and non-histone proteins [4]. Lactylation seems to link metabolic state to gene expression across cardiomyocytes, endothelial cells, fibroblasts, and macrophages, with consequences for contractile function, fibrosis, inflammation, and vascular growth [4,5]. These fuel, signaling, and epigenetic actions intersect and may underline exercise-induced cardio protection while offering new therapeutic entry points. In this review we focus on the role of lactate in cardiovascular health, give a brief summary of the metabolic and signaling functions before outlining the epigenetic effects via lactylation. In addition, we highlight points of convergence between lactates function as fuel, signal and epigenetic modifier, and outline knowledge gaps to guide context-specific, lactate-targeted strategies in cardiovascular disease (CVD).

## Lactate as a metabolic substrate

It is crucial to start by clarifying that lactate should not be confused with lactic acid, as the latter includes a proton. Under normal physiological conditions, lactic acid is not present in the body and the common conception of increased acidity after strenuous exercise is mostly attributed to an increase in protons as a result of ATP breakdown [6,7] while lactate seems to even mitigate an increase in proton concentration [7–9]. Lactate is continuously produced and consumed in normal physiology, both in the form of L-lactate [1] and it’s enantiomer D-lactate [10–12]. Enantiomers are chemical mirror images of each other [13] and in the case of lactate referring to the orientation of the asymmetrical C2-carbon. L-lactate is the predominant form in human physiology and for the remainder of this review lactate refers to the L-enantiomer unless otherwise specified.

### Lactate as a ‘metabolic waste product’

The foundational understanding of lactate’s role in metabolism began with the discovery of the Cori cycle by Carl and Gerty Cori in the 1930 s [14]. This cycle demonstrated how lactate, produced by glycolysis in peripheral tissues, is transported to the liver for conversion back to glucose. This process established lactate as a mere intermediate in energy metabolism, particularly during anaerobic conditions. Subsequently, high circulating lactate levels have been almost exclusively associated with an increased risk of mortality of patients suffering CVD [15–17]. In contrast to this, there are numerous studies that demonstrate the safety and occasionally the efficacy of administering exogenous lactate, e.g., following cardiac stress [18,19], improving cardiac function in heart failure (HF) patients [19,20] and in individuals in intensive care after coronary bypass surgery [21]. In addition, in patients recovering from severe hemorrhagic shock, lactate improves cardiac efficiency in the presence of free fatty acids, potentially through anaplerosis of the tricarboxylic acid cycle [22]. Consequently, it is therefore clear that the role of lactate goes far beyond being a marker for mortality in CVD.

### Lactate as a mitochondrial fuel

More recent research, spearheaded amongst others by Brooks [1] and Magistretti [23], revealed that lactate is not only a byproduct but a critical fuel source for mitochondrial energy production, especially in metabolically active tissue such as the brain, skeletal muscle, and the heart [1]. The heart is a versatile organ with a substantial capacity to oxidize various substrates. Under normal physiological conditions, cardiomyocytes predominantly rely on fatty acid oxidation for energy, which constitutes approximately 40–70% of the total energy supply [24–26], the remainder being mostly carbohydrates [24,27]. Amongst those carbohydrates, lactate is an often-overlooked metabolite which has been demonstrated to be readily oxidized by the heart when available. At normal physiological lactate levels (0.5 mM), lactate is transported into cardiomyocytes and oxidized, accounting for up to 25% of the substrate for oxidation and at elevated physiological levels (4.5 mM), lactate becomes the preferred substrate for the heart compared to glucose and free fatty acids, comprising up to 90% of the total substrates oxidized [28–30]. In vivo studies indicate that most lactate absorbed by the heart is oxidized, and the uptake and oxidation of lactate by the myocardium increases with heightened exercise intensity, exceeding the uptake and oxidation rate of glucose [31].

Lactate is produced during both anaerobic metabolism as well as aerobic glycolysis and will stimulate TCA-cycle dependent oxidative phosphorylation or be used towards building the carbon backbone of macromolecules [32]. Lactate is oxidized to pyruvate via lactate dehydrogenase (LDH) in the cytosol which is then transported into mitochondria via the mitochondrial pyruvate carrier (MPC) [33]. Notably, lactate oxidation seems to persist even in the absence of MPCs in the heart, suggesting alternative pathways for mitochondrial lactate uptake [2,34]. The monocarboxylate transporters (MCTs) have since been identified as principal lactate transporters [1,2,35]. In oxidative tissue, such as the heart, MCT isoforms, MCT1 and MCT4, have been shown to be responsible for lactate uptake and export, respectively. The facilitation of lactate diffusion from glycolytic to more oxidative tissue by MCT isoforms [36–38] allows for lactate to be used by oxidative cells, such as cardiomyocytes [35,39], and what is known as the ‘lactate shuttle’ [40,41] for both intercellular and intracellular lactate transport. Importantly, the discovery of mitochondrial MCTs, as well as mitochondrial MPCs have provided support for intracellular lactate ‘shuttling’ and for direct mitochondrial lactate oxidation [40,42–44].

The extent of lactate ‘shuttling’ within cells is dependent on the concentration gradients created by the mitochondrial respiratory apparatus in recipient cells for oxidative disposal of lactate [40]. In accordance, the mitochondrial lactate oxidation complex (mLOC), a colocalization of MCT1, mitochondrial cytochrome oxidase (COx) and LDH in the mitochondria (mLDH), was shown by Hashimoto et al. to be present in skeletal muscle and other tissues [40,45]. This complex would potentially couple mitochondrial lactate oxidation with mitochondrial redox state [45] and thereby also be important for the regulation of intracellular ROS signaling [4,44,46]. However, this concept is based on the presence of LDH in the mitochondrial matrix, which remains a controversial finding [40]. Therefore, the location of mitochondrial matrix LDH as well as the location of mitochondria lactate oxidation is a topic of ongoing investigation, as this would represent an important regulatory role for lactate in cellular redox [47].

Of the MCT isoforms, MCT2 has been shown to be essential in neural lactate uptake in the context of the astrocyte-neuron lactate shuttle [41], facilitating activity dependent lactate uptake by neurons such as during sleep/wake regulation [48]. Although MCT2 has been demonstrated in cardiac tissue [49], it’s function in cardiac tissue remains poorly described. Conversely, MCT4 which exports lactate from cells, is typically expressed at low levels and also important for the maintenance of intracellular lactate homeostasis [37].

In the heart, MCT isoforms have been shown to play a key role in the development of HF, contributing to a disruption in pyruvate-lactate balance [50,51]. Deletion of MCT1 was associated with increased mitochondrial membrane potential, elevated intracellular calcium and ROS levels, and the acceleration of HF development [2]. The elevation of MCT4, in concert with MPC, was also suggested to be a driving force of cardiac hypertrophy and HF development [52]. As mitochondrial dysfunction is considered a precursor to HF, changes in mitochondrial density, cristae ultrastructure and fewer inter-mitochondrial junctions following loss of MPC activity suggest that mitochondrial lactate uptake may be important to prevent loss of mitochondrial integrity or oxidative stress [52]. MCT4 was also upregulated during diabetic cardiomyopathy and fatty acid oversupply [38]. This was associated with inflammation and oxidative stress in the diabetic heart, whereas upon MCT4 inhibition cellular lactate levels were restored along with mitochondrial membrane potential, ATP levels, and lowered ROS generation. Furthermore, inhibition of lactate efflux in cardiomyocytes restored histone lactylation in co-cultured macrophages and lowered inflammatory response [38].

Although reported to exist in much smaller concentrations than L-lactate, also D-lactate production occurs in humans. It is reported to be mostly produced by gut-bacteria [39,53], but is also produced in mammalian cells where it is derived from methylglyoxal, which is a byproduct of glucose metabolism [54,55]. It may also be produced from pyruvate via D-LDH which has been shown to be present in human mitochondria [56]. Interestingly, D-lactate stimulates oxidative phosphorylation without excessive NADH production, thus lowering the reductive stress when compared to L-lactate [32]. Moreover, D-lactate seems to be readily metabolized in mitochondria [57–60] and even Cori and Cori showed already a century ago a significantly higher uptake of D-lactate vs L-lactate in rats [14], which has been confirmed in humans later as well [58,59]. However, despite those findings, D-lactate seems to be largely dismissed as an energy substrate and is usually associated with pathological states including inflammation [61] mitochondrial dysfunction and oxidative stress [62].

The role of lactate in mitochondrial metabolism, particularly within highly metabolic tissues such as the heart, skeletal muscles, and brain, is gradually gaining recognition as a vital energy substrate. Its contribution is increasingly appreciated, especially during conditions of cardiac stress or hypoxic challenges, where it often proves to be a beneficial fuel source for energy production [29].

## Lactate as a signaling molecule: HCAR1/GPR81

Besides its role as a metabolic fuel, lactate influences a wide array of physiological processes through its interaction with the lactate receptor hydroxycarboxylic acid receptor 1 (HCAR1, also known as HCA1 or GPR81). HCAR1 is an G-protein coupled receptor for L-lactate and has been reported to be present in various tissues, including adipocytes, brain and skeletal muscles [63–65]. So far, there is no published data showing signaling from D-lactate via receptor interaction.

In adipocytes, HCAR1 is known to inhibit lipolysis [66], enabling a switch from fat metabolism to lactate metabolism when lactate levels are high. In skeletal muscle cells HCAR1 signaling has been shown to induce hypertrophy [64], improve mitochondrial maintenance [67] and modulate the uptake of metabolites [68], potentially supporting muscle growth after high-intensity exercise which is known to increase systemic lactate levels dramatically [69]. These effects highlight lactate’s role in coordinating energy metabolism across tissues. In the brain, HCAR1 activation acutely reduces neuronal firing frequency [70] and may thereby attenuate the excitotoxic effect of hypoxia. This is supported by the recent discovery of HCAR1 dependent reduction in glutamate damage in astrocytes [71]. Interestingly, HCAR1 activation seems to trigger an internalization process of the receptor [66] which is common amongst GPCR’s and should not be mistaken for desensitization, but rather the initiation of a long-term signaling process (for review see [72]). Additionally, we and others have shown that HCAR1 activation indeed triggers long-term hypoxia-protective physiological processes: In the brain, lactate injections as well as high-intensity interval training (HIIT) increases vascularization in the brain in wild-type mice but not HCAR1-knockout mice [3]. Furthermore, we and others have shown that HCAR1 promotes neurogenesis and microglia activation [73–75] and we have demonstrated that only two lactate injections 24 and 48 hours after stroke lead to almost 50% reduced lesion size via HCAR1 activation [76]. While lactate treatment doesn’t seem to affect anxiety via HCAR1 [3], the behavioral effects of HCAR1 activation after stroke remain to be elucidated. Collectively, it seems that signaling via HCAR1 mediates a wide array of protective physiological processes in situations of high physiological lactate.

### Potential cardiovascular implications

Although HCAR1’s role in the cardiovascular system remains underexplored, its effects on metabolism, angiogenesis, and neuroprotection suggest potential relevance in similar cardiac processes. For example, lactate stimulated subcutaneous angiogenesis in mice [77] and lactate mediated migration of endothelial cells in culture [78]. Furthermore, lactate-induced angiogenesis via HCAR1 could support vascular repair in ischemic heart disease and HCAR1 might play a role in substrate regulation during times of stress when the heart down-regulates its use of lipids and upregulates lactate-consumption. Databases such as the *UCSC Cell Browser* for humans or *Tabula Muris* for mice suggest HCAR1 being predominantly located on fibroblasts and endothelial cells in the heart. This raises the intriguing possibility that HCAR1 activation may influence cardiac fibroblast function, particularly given their critical role in extracellular matrix remodeling and signaling within the heart [79,80].

## Lactate as an epigenetic modifier: lactylation

In addition to the metabolic and signaling role of lactate, Zhang et al. reported in 2019 for the first time the process of lactylation and thereby a role of lactate in post-translational protein modification [4]. Since this initial description, a constantly growing number of lactylated proteins and histone lactylation sites have been described, as summarized in a recent review by Hu et al. [81].

**Figure 1.**
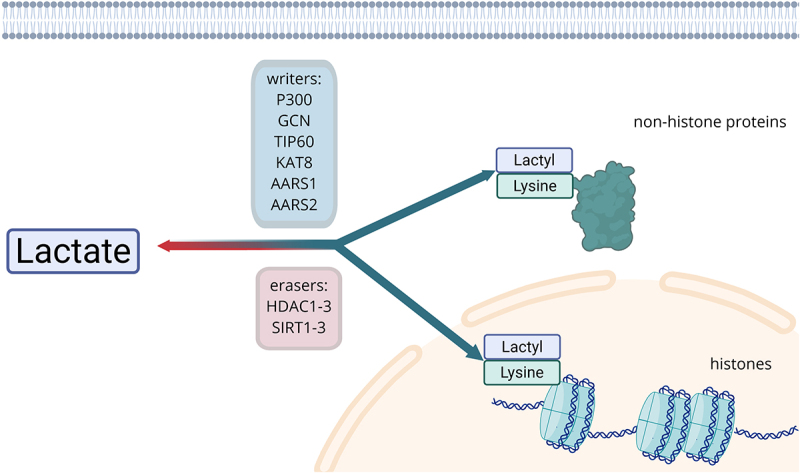
Cytosolic lactate can act as an epigenetic modifier under action of proteins recognised as writers, leading to attachment of lactyl group to lysine residues, which was found to occur in histones and other proteins involved in various processes. The marking can be reversed by action of eraser proteins. Image created with BioRender.com.

Similar to other post-translational modifications processes such as acetylation or methylation [82], during lactylation, a lactyl group is added covalently to specific areas of proteins, in this case lysine residues, thereby modifying its behavior [4,83] (Figure 1). This process starts with the formation of lactyl groups by catabolism of extracellular lactate transported into the cytoplasm through MCTs, but also from lactate derived from glucose metabolism. These lactyl groups are then transferred to lysine residues of substrate proteins (histone and non-histone proteins) by the action of ‘writer proteins’ such as P300 [4], GCN5 [84], TIP60 [85], KAT8 [86], AARS1, and AARS2 [87]. As other post-transcriptional modifications, lactylation is not permanent, and can be removed by ‘eraser proteins’ like HDAC1-3 and SIRT1-3 [88].

Lactylation and other histone acylations, like for example acetylation, acetoacetylation, and, benzoylation, make chromatin more open and accessible. In fact, CHiP-seq data shows co-occurrence of H3K27ac and lactylation in promoter and distal areas where also the active or poised region marks H3K4me3 and H3K4me1 can be detected [89]. Therefore, histone lactylation can stimulate gene transcription [4] and since its discovery, it has been implicated in a plethora of biological and pathological processes such as embryonic development [89], inflammation [90], cancer [91], senescence [92] and CVD [93,94]. Interestingly, the lactylation process is mostly reported to have a protective effect in pathological settings. In this section, we focus on the most prominent reports that have been uncovering the role of lactylation in the cardiovascular system and that are summarized in Table 1.

**Table 1.** Lactylation in the heart and vasculature.

Lactylation site	Cardiovascular cell type	Role	Reference
	**Histone**		
H3K9	Endothelial cells	Angiogenesis induction	Fan, Zeng et al. [95]
H3K18	Cardiomyocytes	Promotes hypertrophy	Zhao, Liu et al. [96]
	Monocytes	Reparative transcription in MI	Wang, Wang et al. [84]
	Vascular smooth muscle cells	Inhibiting HIF1α	Ma, Jia et al. [94]
H4K12	Vascular smooth muscle cells	Inducing senescence	Li, Chen et al. [97]
	**Non-histone**		
SA3K-K351	Fibroblasts	Activation of cardioprotective proteins	Wang, Li et al. [93]
α-MHC-K1897	Cardiomyocytes	Sarcomere stabilization in HF	Zhang, Zhang et al. [83]
Mecp2-K271	Endothelial cells	Suppression of atherosclerosis	Wang, Chen et al. [98]

### Lactylation in cardiovascular development

Histone lactylation decreases after birth in the mouse heart, while non-histone lactylation increases, suggesting distinct roles during development [99]. Interestingly, increased lactylation is observed after birth in non-histone proteins regulating crucial cardiomyocyte functions like energy metabolism (GAPDH, Atp5f1b, Sdha, etc.) or myocardial contraction (Myh7, Myl2, Ttn, etc.) [99]. On the other hand, loss of lactylation in genes like *E2f2* and *Rfc3* correlates with reduced cell proliferation, highlighting its potential importance during early cardiac maturation by regulating cardiomyocyte cell cycling exit [99]. On the basis of these findings, lactylation driven transcription of genes such as E2f2 and Rfc3 may also be a potential therapeutic target for cardiomyocyte regeneration post-myocardial infarction (MI).

### Lactylation in cardiac disease

The heart is formed by a variety of cell types. These different cells perform different functions and the effects of lactylation vary from one cell type to the other, therefore contributing differently to the development of cardiac disease.

#### Cardiomyocytes

Thanks to a very specific set of features, cardiomyocytes can contract during the whole lifespan of an individual, with very low cell turnover, even upon injury. One of the main reasons for this slow turnover and reduced proliferation ability is the extremely efficient and complex contractile apparatus, which makes its disassembly extremely complicated [100]. Interestingly, increased lactylation (K1897) of α-MHC stabilizes sarcomeres and improves cardiac function in a mouse model of HF [83]. Moreover, histone lactylation in cardiomyocytes (H3K18la) promotes hypertrophy, which can be reversed by reducing lactate levels *in vitro* [96]. While the authors argue for pathological hypertrophy, it cannot be excluded that histone lactylation may also promote physiological hypertrophy [101,102], especially keeping in mind the angiogenic effect of lactate both via HCAR1 signaling and lactylation.

#### Fibroblasts

Fibroblasts are essential in creating and maintaining the extracellular matrix, i.e., the scaffold that sustains the heart, which affects cardiac development and disease via paracrine signaling during the postnatal period [79,80]. In this context, lactylation (K351la) of Serpina3K in fibroblasts protects cardiomyocytes from ischemia-reperfusion injury via activation of the cardioprotective proteins RISK and SAFE [93]. A key role for cardiac fibroblast is the deposition of extracellular matrix proteins like FN1 or COL1a1 to form the scar which stabilizes the heart after MI [103]. However, in order to do so, cardiac fibroblasts must first change to an activated phenotype.

Interestingly, histone lactylation is observed in activated fibroblasts from kidney (H4K12la) [104] after ischemia, and also from the lung (H3K18la) [105] as a consequence of arsenate exposure. While the role of histone lactylation during fibroblast activation in the heart remains unclear, data from other tissues suggest that this could be a pan-fibroblast mechanism.

#### Macrophages

The more prominent role of macrophages is to phagocytose dying cells, debris, and other waste products. In addition, macrophages secrete signaling factors which are important during the healing process. These cells also exhibit a great diversity of expression profiles, as every tissue has its own specialized resident macrophages [106]. Macrophages were the first cells where histone lactylation was described [4], and since then numerous studies have described the role of lactylation in macrophages, especially during polarization, as recently reviewed by Bao et al. [107]. In the context of MI, histone lactylation (H3K18la) has been shown to promote a reparative transcriptional response in monocytes, which upon arrival to the site of cardiac injury transition into macrophages with pro-angiogenic and anti-inflammatory activities [84]. Furthermore, the upregulation of MCT4 and concomitant lactate efflux in diabetic cardiomyopathy model have been associated with histone H4K12 lactylation in cardiac infiltrating macrophages [38]. Interestingly, the polarization effect of histone lactylation (H3K18la) towards a reparative (M2) phenotype has also been described in skeletal muscle tissue [108].

Mitochondrial fission and fusion are adaptations that can contribute to meet the metabolic demands of the cell [109,110]. Recent evidence demonstrates that mitochondrial fission and fusion may also produce specific signals to other organelles to dictate cell differentiation and cell phenotype [111,112], shown to be important during ischemic injury [113] as well as during heart failure with preserved ejection fraction [114]. In macrophages, mitochondrial fragmentation was associated with increased lactate and histone lactylation, and shown to be an important step in the resolution phase of inflammation [115]. In line with this, mitochondrial dynamics may have an important role in controlling metabolite levels such as lactate [115]. Similar findings are described in kidney cells, where during sepsis-induced kidney injury the downregulation of pyruvate dehydrogenase (PDH) resulted in lactate overproduction. This in turn mediated the lactylation of Fis1 promoting excessive mitochondrial fission, and was associated with lower ATP levels, increased mt-ROS and apoptosis [116]. Finally, lactylation by D-lactate (K310) has been also reported to attenuate inflammatory signaling and NF-κB transcriptional activity to restore immune homeostasis [90].

### Lactylation in vascular disease

#### Angiogenesis

Lactylation (H3K9la) of a set of pro-angiogenic genes like *Nectin1*, *Tgfbr2*, and *Lam4*, inhibits the expression of the lactylation eraser *Hdca2* and has been reported to induce angiogenesis in endothelial cells [95]. Besides histones, lactylation (K183la) of transcription factors like YY1 in the ocular nerve microglia has been implicated in enhanced FGF2 transcription, which increases angiogenesis [117]. Interestingly, YY1 lactylation also involves p300, and blocking these proteins using small molecules also reduces angiogenesis [117], making p300 an interesting therapeutic target. In the context of cancer, hypoxia triggers also angiogenesis through the HIF1α-VEGF-SEMA3A axis. Supporting this, lactylation of HIF1α has been shown to increase angiogenesis through KIAA1199 enhanced transcription [118]. Building on this, the use of the natural alkaloid evodiamine has been shown to reduce angiogenesis through inhibiting HIF1α histone lactylation (H3K18la). Therefore, inhibition of lactylation via p300 or in the context of hypoxia, of HIF1α histone lactylation, establishes an interesting precedent to therapeutically intervene angiogenesis.

#### Atherosclerosis

A key mechanism during the formation of foam cells are macrophages that ingest lipids, giving them a foamy appearance. These cells form the plaque that leads to atherosclerosis [119]. As mentioned for cardiac macrophages, there is a growing body of data that implicates lactylation in reparative macrophage polarization, protecting from atherosclerosis [120,121]. Specifically, reduced intracellular lactate and H3K18 lactylation increase the expression of pro-inflammatory genes in macrophages, increasing lipid ingestion, while MCT4 (efflux lactate transporter) deficiency, increases lactylation and polarization towards M1-reparative phenotype [120]. In addition, K271-lactylation of the transcriptional repressor MECP2 has been also shown to promote M2 macrophage polarization in atherosclerosis, reducing plaque area and shrinking necrotic cores.

In addition, endothelial cells have also a crucial role in the development of atherosclerosis. Interestingly, in a mouse model of atherosclerosis, exercise suppressed the development of plaque by increasing lactylation (K271la) of Mecp2 in endothelial cells [98]. Furthermore, it was found that the suppression of atherosclerosis was triggered by lactylated Mecp2 repression of *Ereg*, which altered the MAPK signalling pathway [98]. Finally, another important player in atherosclerosis is vascular smooth muscle cell senescence. TNFα is well reported to induce cell senescence [122–124] and its receptor TRAP1 has been shown to induce vascular smooth muscle cell senescence and a senescence secretory phenotype via increased histone lactylation (H4K12la) [97]. Furthermore, specific knock-out of *Trap1* in vascular smooth muscle cells attenuated atherosclerosis development *in vivo* [97]. Reduced lactate production and lactylation have been reported to progress vascular smooth muscle calcification, that have been attributed to the effect of NR4A3, an orphan nuclear receptor [94]. Together these findings reflect the importance of lactylation in atherosclerosis.

#### Endothelial to mesenchymal transition (EndoMT)

During development and disease, endothelial cells can activate EndoMT, causing loss of endothelial cell features while gaining a mesenchymal cell phenotype. For example, fibrosis is associated EndoMT [125], and the pro-EndoMT factor *Snail1* is known to be lactylated through p300 during this process [126]. Supporting these findings, lactylation (H3K18la) of *Snail1* through p300 has been also implicated in EndoMT in the context of atherosclerosis [127]. Furthermore, lactylation (HK150la) through p300 of an additional important regulator of EndoMT, Twist1, has been described to increase fibrosis in flap transplantation [128]. Together, these findings support a pro-EndoMT gene expression activation through lactylation, which is of great relevance in the field of fibrosis.

#### Hypertrophy

On the topic of cardiac hypertrophy, it is important to distinguish between physiological and pathological hypertrophy. Physiological hypertrophy is triggered by increased physiological demands such as exercise or pregnancy. The process is adaptive and reversible and coordinated with proportional angiogenesis and preserved/increased function [129,130]. Pathological hypertrophy is triggered by events such as cardiac overload, MI or diabetes, leading initially to compensatory effects but results eventually decreased heart-function or failure due to fibrosis, chamber dilatation, arrythmia and more [129,130]. Pathological hypertrophy often generates a pro-lactylation milieu such as high glycolytic flux, cytosolic lactate levels, MCT4 upregulation and impaired MPC/PDH, hence increasing substrate availability for histone/non-histone lysine lactylation. On the other hand, physiological stimuli (exercise/pregnancy) transiently elevate lactate in a setting of intact mitochondrial function, NO signaling, and coordinated angiogenesis and is more likely to channel lactate toward adaptive signaling and selective lactylation [129].

In conclusion, it seems that the effect of lactylation is cell type-dependent and a broad activator or inhibitor of lactylation might not be an effective therapeutical approach. On the other hand, lactylation could be used as a biomarker and its target proteins could be specifically targeted as a therapeutic approach.

## Interconnected mechanisms

### Role of MCTs in regulating lactate availability

MCTs appear to be essential for controlling lactate transport across membranes and controlling its availability for both metabolism, signaling and lactylation. MCT1/2-mediated lactate import supports mitochondrial ATP production, while MCT4-mediated export prevents intracellular lactate accumulation, which could drive excessive lactylation. Future studies should explore the role of MCTs in regulating lactate availability across different tissues and disease contexts. Key areas of focus may include understanding how MCT1/2 and MCT4 influence lactate-driven processes such as mitochondrial function, HCAR1 signaling, and histone/non-histone lactylation, as well as their interplay with metabolic pathways under stress conditions like hypoxia or exercise. Investigating tissue-specific roles of MCTs in cardiomyocytes, fibroblasts, and macrophages, along with their dysregulation in diseases such as HF, diabetic cardiomyopathy, and cancer, could reveal therapeutic potential. Additionally, targeting MCTs to restore lactate homeostasis or modulate lactate-driven epigenetic and signaling processes may offer novel strategies for treating metabolic and cardiovascular diseases.

### Lactylation and cardiac mitochondria

MRPL3 mitochondrial ribosomal protein L3 was found to play a role in hypertrophic cardiomyopathy and help with protein synthesis in the mitochondria [131]. MRPL3 was linked with HIBCH, an enzyme vital for mitochondrial amino acid metabolism, suggesting a cooperative role in regulating mitochondrial and lactylation-mediated metabolic pathways. Experimental validation confirmed that silencing MRPL3 disrupted mitochondrial function and inhibited hepatocellular carcinoma cell proliferation, migration, and invasion. These findings suggest that MRPL3 not only contributes to mitochondrial metabolism but also integrates lactylation-related epigenetic regulation, underpinning its critical role in hepatocellular carcinoma pathophysiology [132]. A possible role for MRLP3 during lactylation has not been shown in the heart yet, and suggests an interesting topic for future research.

### Fibroblasts as a central node

Cardiac fibroblasts are involved in both lactate signaling and lactylation [93]. HCAR1 activation in fibroblasts may regulate extracellular matrix remodeling, while histone lactylation could drive fibroblast activation during fibrosis, as reported in other organs like the lung or the kidney [104,105]. Investigating the interplay between HCAR1 signaling and lactylation in fibroblasts could reveal novel mechanisms for fibroblast activity during cardiac fibrosis. Of particular interest would be to explore whether lactylation is involved in general fibroblasts activation or is involved in specific processes such as post-MI scar formation or perivascular fibrosis.

## Potential therapeutic interventions on lactate and lactylation: who, when, and where

High lactate levels after MI are still regarded as a prognostic tool at best, indicating an increased risk for mortality. Considering the promising results from lactate injections after MI, further studies should be conducted to determine the detailed mechanisms of lactate actions during/post-MI. Its prominent role as cardiac fuel during stress might provide cellular energy when it is needed most. Post-MI, pharmaceutical activation of HCAR1 might accelerate cardiac repair and increase coronary vascularization, ameliorating the risk for subsequent MI.

Regarding lactylation, possible therapeutic approaches would require a cell type-specific approach. For example, a controlled reduction of cardiac fibroblast activation through inhibition of lactylation could lead to smaller scars post-MI, and less cardiac fibrosis, which would prevent or slow down the progression of HF. Contrarily, therapeutics aiming to increase lactylation of α-MHC in cardiomyocytes might help to limit the progression of HF. In this case, increased lactylation in CM should be targeted to α-MHC as histone lactylation has been shown to induce physiological hypertrophy. Finally, in the case of macrophages, histone lactylation seems to induce polarization towards a reparative phenotype rather than an inflammatory phenotype, which could be beneficial in a broader spectrum of situations like atherosclerosis and MI. All this could be achieved by targeting possible cell-specific proteins involved in the lactylation process, but to find such targets, further research is paramount.

## Conclusions

Contrary to the outdated mantra, lactate is far more than a metabolic byproduct; it is a versatile molecule that integrates metabolism, signaling, and epigenetics, and may be an important regulator of cardiovascular health. Its role as an important carbon source, transport via MCTs, signaling through HCAR1, and modification of proteins via lactylation are interconnected mechanisms that influence many physiological processes including cardiac and vascular function. Understanding these connections will be critical for developing lactate-based therapies for CVD. Notably, most of lactate’s effects can contribute towards protection in regard to exercise/hypoxia and by extension also in the context of HF. As discussed above, lactate is an important energy source in the stressed heart and exercising muscles, it contributes to increased vascularization, ameliorates damage after HF and more. Lactate could therefore be a key driver of exercise-induced cardiovascular health and a potential tool to treat HF patients. The challenge for future research will be to dis-entangle the interconnected effects of lactate on metabolism, signaling via HCAR1 and both histone and non-histone lactylation.

## Data Availability

No new data was generated for this review.

## List of abbreviations

α-MHC: myosin heavy chain, α isoform
AARS1: alanyl—tRNA synthetase 1
AARS2: alanyl—tRNA synthetase, mitochondrial
ATP: adenosine Triphosphate
Atp5f1b: mitochondrial ATP synthase subunit gene
CHiP-seq: chromatin immunoprecipitation
COL1a1: collagen, type I, alpha 1
CVD: cardiovascular disease
*E2f2*: transcription factor E2F2 gene
EndoMT: endothelial to mesenchymal transition
*Ereg*: epiregulin gene
FGF2: fibroblast growth factor 2
FN1: fibronectin 1
GAPDH: glyceraldehyde 3-phosphate
GCN5: general control non-repressed 5 histone
GPCR: G-protein-coupled receptor
GPR81: G-protein-coupled receptor 81
H: histone, followed by a number indicating
HCAR1: hydroxycarboxylic acid receptor 1
HDAC1: histone deacetylase 1
*Hdca2*: histone deacetylase 2 gene
HF: heart failure
HIBCH: 3-hydroxyisobutyryl-CoA hydrolase
HIF1α: hypoxia inducible factor1-alpha
K: lysine, followed by a number indicating
KAT8: lysine acetyltransferase 8
*Lam4*: laminin subunit alpha-4 gene
LDH: lactate dehydrogenase
M1: pro-inflammatory activated macrophage
M2: anti-inflammatory activated
MCT: monocarboxylate transporters
Mecp2: methyl CpG binding protein 2 gene
MECP2: methyl CpG binding protein 2
MI: myocardial infarction
MPC: mitochondrial pyruvate carrier
MRPL3: mitochondrial ribosomal protein
Myh7: beta-myosin heavy chain
Myl2: myosin light chain 2, ventricular gene
NADH: nicotinamide adenine dinucleotide plus
NO: nitric oxide
NR4A3: nuclear receptor 4A3
p300: histone acetyltransferase p300
PDH: pyruvate dehydrogenase
*Rfc3*: replication factor C subunit 3 gene
RISK: reperfusion injury salvage kinase pathway
ROS: reactive oxygen species
SA3K: serpina3K
SAFE: survivor activating factor enhancement pathway
Sdha: succinate dehydrogenase complex, subunit A,
SEMA3A: semaphorin-3A
SIRT1: sirtuin 1
*Snail1*: zinc-finger transcription factor gene
*Tgfbr2*: transforming growth factor-beta receptor type I gene
TIP60: histone acetyltransferase KAT5
*Trap1*: tumor necrosis factor receptor-associated protein 1
Ttn: titin gene
VEGF: vascular endothelial growth factor
